# Supplementary material for: TGF-β-3 Induces Different Effects from TGF-β-1 and -2 on Cellular Metabolism and the Spatial Properties of the Human Trabecular Meshwork Cells
Source: Int J Mol Sci. 2023 Feb 20;24(4):4181. doi: 10.3390/ijms24044181 (PMC9960590; doi:10.3390/ijms24044181)
Supplement: Supplementary file 1 [file ijms-24-04181-s001.zip › ijms-2191692-supplementary.pdf]

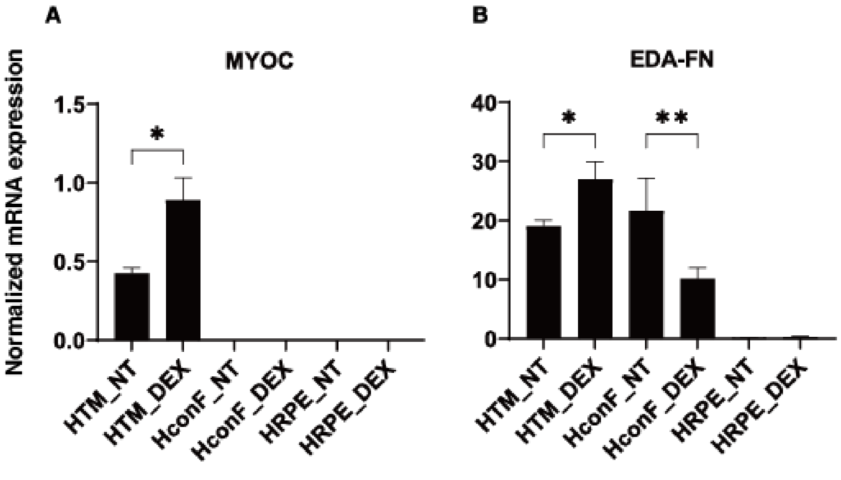

Supplemental Figure S1. mRNA expression of myocilin or EDA fibronectin in 2D cultured HTM, human conjunctival fibroblast (HconF) or human retinal pigment epithelium (HRPE).

To ensure that these HTM cells are truly TM cells, the DEX induced up-regulation in the mRNA expression of myocilin and extra domain A (EDA) fibronectin was confirmed among the criteria described in the consensus recommendations for TM cells as described by Keller et al.[63]. As negative controls, human conjunctival fibroblasts (HconFs, ScienCell Reserch laboratories, CA USA) and human retinal pigment-epithelium (HRPE, ATCC, VA USA) were also used.

In the absence or presence of 250 nM DEX, 2D cultured HTM, HconF or HRPE were subjected to qPCR analysis at Day 6 to estimate the expression of mRNA of myocilin (*MYOC*, panel A) or EDA fibronectin (*EDA-FN*, panel B). All experiments were performed in duplicate using fresh preparations. Data are presented as the arithmetic mean ± the standard error of the mean (SEM). * $P<0.05$, ** $P<0.01$ (ANOVA followed by Tukey's multiple comparison test).

Supplemental Table S1. Sequences of primers of qPCR

		Sequence	Exon Location	RefSeq Number	Product Length (bp)
human RPLP0*1	Probe	5'-/56-FAM/CCCTGTCTT/ZEN/CCCTGGGCATCAC/3IABkFQ/-3'	2-3	NM_001002	143
	Primer2	5'-TCGTCTTTAAACCCTGCGTG-3'			
	Primer1	5'-TGTCTGCTCCCACAATGAAAC-3'			
human COL1A1*1	Probe	5'-/56-FAM/TCGAGGGCC/ZEN/AAGACGAAGACATC/3IABkFQ/-3'	1-2	NM_000088	115
	Primer2	5'-GACATGTTCAGCTTTGTGGAC-3'			
	Primer1	5'-TTCTGTACGCAGGTGATTGG-3'			
human COL4A1*1	Probe	5′-/56-FAM/TCATACAGA/ZEN/CTTGGCAGCGGCT/3IABkFQ/-3′	51-52	NM_001845	142
	Primer2	5′-AGAGAGGAGCGAGATGTTCA-3′			
	Primer1	5′-TGAGTCAGGCTTCATTATGTTCT-3′			
human COL6A1*1	Probe	5′-/56-FAM/CAGGTTTCG/ZEN/GTCACAGCGGTAGT/3IABkFQ/-3′	2-3	NM_001848	114
	Primer2	5'-CCTCGTGGACAAAGTCAAGT-3'			
	Primer1	5'-GTGAGGCCTTGGATGATCTC-3'			
human FN1*1	Probe	5′-/56-FAM/TACAGCTTA/ZEN/TTCTCCCTCGCCCAG/3IABkFQ/-3′	3-4	NM_212482	129
	Primer2	5'-CGTCCTAAAGACTCCATGATCTG-3'			
	Primer1	5'-ACCAATCTTGTAGGACTGACC-3'			
human αSMA*1	Probe	5'-/56-FAM/AGACCCTGT/ZEN/TCCAGCCATCCTTC/3IABkFQ/-3'	8-9	NM_001613	105
	Primer2	5'-AGAGTTACGAGTTGCCTGATG-3'			
	Primer1	5'-CTGTTGTAGGTGGTTTCATGGA-3'			
human TIMP1*1	Probe	5′-/56-FAM/TCAACCAGA/ZEN/CCACCTTATACCAGCG/3IABkFQ/-3′	2-4	NM_003254	121
	Primer2	5′-CCTTCTGCAATTCCGACCT-3′			
	Primer1	5′-GCTTGGAACCCTTTATACATCTTG-3′			
human TIMP2*1	Probe	5′-/56-FAM/TCTCATTGC/ZEN/AGGAAAGGCCGAGG/3IABkFQ/-3′	3-4	NM_003255	133
	Primer2	5′-GACGTTGGAGGAAAGAAGGA-3′			
	Primer1	5′-TGTGGTTCAGGCTCTTCTTC-3′			
human TIMP3*1	Probe	5′-/56-FAM/CCTCCTTTA/ZEN/CCAGCTTCTTCCCCAC/3IABkFQ/-3′	1-3	NM_000362	112
	Primer2	5′-CCTTCTGCAACTCCGACATC-3′			
	Primer1	5′-CGGTACATCTTCATCTGCTTGA-3′			
human TIMP4*1	Probe	5′-/56-FAM/ACTGAGGAC/ZEN/CTGACCAGTCAAGAGA/3IABkFQ/-3′	3-4	NM_003256	149
	Primer2	5′-GGTTTGAGAAAGTCAAGGATGTTC-3′			
	Primer1	5′-GTTGCACAGATGGATGAAGAC-3′			
human MMP2*1	Probe	5′-/56-FAM/TTCTGTCCC/ZEN/CATGAAGCCCTGTTC/3IABkFQ/-3′	6-7	NM_004530	140
	Primer2	5′-TCCACCACCTACAACTTTGAG-3′			
	Primer1	5′-GTGCAGCTGTCATAGGATGT-3′			
human MMP9*1	Probe	5′-/56-FAM/CCAGGAGGA/ZEN/AAGGCGTGTGC/3IABkFQ/-3	3-4	NM_004994	123
	Primer2	5′-ACATCGTCATCCAGTTTGGTG-3′			
	Primer1	5′-CGTCGAAATGGGCGTCT-3′			
human MMP14*1	Probe	5′-/56-FAM/TTGTTCCTC/ZEN/AAAGTGCCTGTTTGCTC/3IABkFQ/-3	1-1	NM_004995	114
	Primer2	5′-TTCGCCGACTAAGCAGAAG-3′			
	Primer1	5′-CTTGAATTCCTAGACCGCTGT-3′			

Supplemental Methods

TEER and FITC dextran permeability measurements of 2D cultured HTM monolayers

The TEER and FITC dextran permeability measurements of HTM cell monolayers were carried out according to previously described methods[56, 57]. In brief, 2D HTM cell monolayer was prepared using 12 well plates for TEER (0.4 μm pore size and 12 mm diameter; Corning Transwell, Sigma-Aldrich) in the absence or presence of 1, 5 or 10 ng/mL TGFβ-1, -2 or -3 and cultured as above. The TEER (Ωcm^2) values were measured at Day 6 using an electrical resistance system (KANTO CHEMICAL CO. INC., Tokyo, Japan) according to the manufacturer's instructions. Alternatively, for measuring FITC-dextran permeability, 50 μmol/L of FITC-dextran (Sigma-Aldrich) was added to the well basal compartments of the culture and the culture medium from the apical compartment was collected after 60 min. The concentrations of the FITC-dextran were measured using a multimode plate reader (Enspire; Perkin Elmer, MA USA) at an excitation wavelength of 490 and an emission wavelength of 530 nm.

Analysis of real-time cellular metabolism of the 2D-cultured HTM cells by a Seahorse Bioanalyzer.

As a real-time cellular metabolic function analysis, the oxygen consumption rate (OCR) and the extracellular acidification rate (ECAR) of the 2D-cultured HTM cells in the absence or presence of the three TGF-β isoforms were simultaneously measured using a Seahorse XFe96 Bioanalyzer (Agilent Technologies, Santa Clara, CA, U.S.A.) as described in a recent report [34, 58, 59]. In brief, 20,000 2D-cultured HTM cells were placed in wells of a 96-well assay plate. After replacing the culture medium with Seahorse XF DMEM assay medium (pH 7.4, Agilent Technologies, #103575-100) supplemented with 5.5 mM glucose, 2.0 mM glutamine, and 1.0 mM sodium pyruvate, the OCR and ECAR values were determined using a Seahorse XFe96 Bioanalyzer at the baseline and thereafter, the samples were further analyzed after sequential injections of 2.0 μM oligomycin, 5.0 μM carbonyl cyanide-p-trifluoromethoxyphenylhydrazone (FCCP), 1.0 μM rotenone and antimycin A, and 10 mM 2-deoxy-D-glucose (2DG). The OCR and ECAR values were normalized to the amount of protein assessed by BCA protein assay kit (TaKaRa, Japan) per well after completion of the assay.

Quantitative PCR

Total RNA extraction followed by reverse transcription and real-time PCR, and the quantification of respective genes normalized by comparing with the expression of a housekeeping gene 36B4 (*Rplp0*) were described previously[29]. Sequence information regarding the primers and Taqman probes used in the present study are shown in Supplemental Table 1.

Immunocytochemistry of 2D cultures of HTM cells and 3D HTM spheroids

Immunocytochemistry of 2D cultured HTM cells and 3D HTM spheroids was examined by previously described methods, with minor modifications[62],[63]. All procedures were performed at room temperature unless otherwise stated. Briefly, 2D HTM cells were cultured on glass slides (Lab-Tek II Chamber slide, Thermo Fisher Scientific Inc.) or 3D HTM spheroids prepared as described above under several experimental conditions were fixed in 4 % paraformaldehyde overnight in PBS, blocked in 3 % BSA in PBS for 3 hours, washed twice with PBS for 30 minutes. These were then reacted with an anti-human COL1 (#600-401-103-0.1, Rockland antibodies &assays, Limerick, PA U.S.A.), COL4 (#600-401-108-0.1, Rockland antibodies &assays, Limerick, PA U.S.A.), COL6 (#600-401-106-0.1, Rockland antibodies &assays, Limerick, PA U.S.A.) or FN (#sc-8422, Santa Cruz Biotechnology, INC., Dallas, TX U.S.A.) rabbit antibody (1:200 dilutions) at 4°C overnight. After washing 3 times with PBS for 1 hour each, they were then reacted with a 1:1000 dilution of a goat anti-rabbit IgG (488 nm, # A-11008, ThermoFischer Scientific, Waltham, MA U.S.A.), phalloidin (594 nm, #20553, Cyman Chemical, Ann Arbor MI U.S.A.) and DAPI (#28718-90-3, Dojindo, Kumamoto, Japan) for 3 hrs, and thereafter mounted with ProLong Gold Antifade Mountant with a cover glass. Immunofluorescent images were obtained by means of a Nikon A1 confocal microscope using a ×20 air objective with a resolution of 1024 × 1024 pixels. To quantify the stain intensities of each target molecule of the 2D cultured cells, 1) their stain intensities among the observed areas were evaluated using Image J (NIS-Elements 4.0 software), 2) numbers of nuclei stained by DAPI within those areas were counted, 3) the stain intensities were divided by the numbers of nuclei, and 4) the relative intensities of each experimental condition were calculated by comparing with those of the control. For 3D spheroids, serial-axis images with a 2.2 μm interval at a height of 35 μm from their surface were obtained. The maximum intensity/surface area among the above observed areas was calculated using Image

J (NIS-Elements 4.0 software) as follows: surface area=D×A/(A+π×H2), where D (μm) indicates spheroid diameter, A (μm^2) indicates the area of the sectioned spheroid, and H (μm) indicates the height (=35 μm). For estimating the numbers of cells within a 3D spheroid, the volume of a 3D spheroid and the volume of a representative cell was calculated by assuming a spherical shape and the tentative diameters were estimated by largest cross-section of phalloidin images of the 3D spheroid (n=5) and the distance between two adjacent nuclei stained by DAPI (n=5 for one section and was repeated five times using different preparations), respectively. The relative intensities of each experimental condition were calculated by comparing with those of the control.

Characterization of the physical properties, sizes and stiffness, of the 3D HTM spheroids

The configuration of the 3D HTM spheroids was observed by phase contrast microscopy (PC, Nikon ECLIPSE TS2; Tokyo, Japan), and the mean size of each 3D organoid that was measured was defined using the largest cross-sectional area (CSA) of the PC image was analyzed using the Image-J software version 1.51n (National Institutes of Health, Bethesda, MD) as described previously [55, 61].

The solidity of the 3D spheroids was measured using a micro-squeezer (MicroSquisher, CellScale, Waterloo, ON, Canada) equipped with a microscale compression system composed of a 406 μm diameter cantilever as recently reported[55]. A single spheroid placed on a 3-mm x 3-mm plate was compressed to a deformation of 50%, as determined by a microscopic camera, for 20 sec. The force required to achieve a 50 % strain was measured through the cantilever, and the data are expressed as force/displacement (μN/μm).
